# Knowledge translation of research findings

**DOI:** 10.1186/1748-5908-7-50

**Published:** 2012-05-31

**Authors:** Jeremy M Grimshaw, Martin P Eccles, John N Lavis, Sophie J Hill, Janet E Squires

**Affiliations:** 1 Department of Medicine, Clinical Epidemiology Program, Ottawa Hospital Research Institute, University of Ottawa, 501 Smyth Road, Box 711, Ottawa, ON, K1H 8L6, Canada; 2 Newcastle University, Institute of Health and Society, Baddiley-Clark Building, Richardson Road, Newcastle upon Tyne, NE2 4AX, UK; 3 Department of Clinical Epidemiology and Biostatistics; and Department of Political Science, McMaster Health Forum, Centre for Health Economics and Policy Analysis, McMaster University, Hamilton, ON, Canada; 4 Centre for Health Communication and Participation, Australian Institute for Primary Care & Ageing, La Trobe University, Bundoora, VIC, 3086, Australia; 5 Clinical Epidemiology Program, Ottawa Hospital Research Institute, Ottawa, ON, Canada

## Abstract

**Background:**

One of the most consistent findings from clinical and health services research is the failure to translate research into practice and policy. As a result of these evidence-practice and policy gaps, patients fail to benefit optimally from advances in healthcare and are exposed to unnecessary risks of iatrogenic harms, and healthcare systems are exposed to unnecessary expenditure resulting in significant opportunity costs. Over the last decade, there has been increasing international policy and research attention on how to reduce the evidence-practice and policy gap. In this paper, we summarise the current concepts and evidence to guide knowledge translation activities, defined as T2 research (the translation of new clinical knowledge into improved health). We structure the article around five key questions: what should be transferred; to whom should research knowledge be transferred; by whom should research knowledge be transferred; how should research knowledge be transferred; and, with what effect should research knowledge be transferred?

**Discussion:**

We suggest that the basic unit of knowledge translation should usually be up-to-date systematic reviews or other syntheses of research findings. Knowledge translators need to identify the key messages for different target audiences and to fashion these in language and knowledge translation products that are easily assimilated by different audiences. The relative importance of knowledge translation to different target audiences will vary by the type of research and appropriate endpoints of knowledge translation may vary across different stakeholder groups. There are a large number of planned knowledge translation models, derived from different disciplinary, contextual (*i.e.*, setting), and target audience viewpoints. Most of these suggest that planned knowledge translation for healthcare professionals and consumers is more likely to be successful if the choice of knowledge translation strategy is informed by an assessment of the likely barriers and facilitators. Although our evidence on the likely effectiveness of different strategies to overcome specific barriers remains incomplete, there is a range of informative systematic reviews of interventions aimed at healthcare professionals and consumers (*i.e.*, patients, family members, and informal carers) and of factors important to research use by policy makers.

**Summary:**

There is a substantial (if incomplete) evidence base to guide choice of knowledge translation activities targeting healthcare professionals and consumers. The evidence base on the effects of different knowledge translation approaches targeting healthcare policy makers and senior managers is much weaker but there are a profusion of innovative approaches that warrant further evaluation.

## Background

Globally we spend billions of dollars each year in both the public and private sectors on biomedical, clinical, and health services research, undergraduate healthcare professional training and continuing professional development, quality improvement, patient safety, and risk management. Despite this, healthcare systems fail to ensure that effective and cost-effective programs, services, and drugs get to all of those who need them; and healthcare professionals fail to provide the level of care to which they aspire. One of the most consistent findings from clinical and health services research is the failure to translate research into practice and policy. For example, McGlynn and colleagues observed that patients in the USA received 55% of recommended care, and that quality varied by medical condition ranging from 79% of recommended care for senile cataract to 11% of recommended care for alcohol dependence [1]. Similar findings have been reported globally in both developed and developing settings, in both primary care and specialty-provided care and in care provided by all disciplines [2]. As a result of these evidence-practice gaps, patients fail to benefit optimally from advances in healthcare resulting in poorer quality of life and loss of productivity both personally and at the societal level.

In addition to the limited use of effective treatments, there is also evidence that around 20% to 30% of patients may get care that is not needed or care that could be potentially harmful [3]. Because of these evidence-practice gaps, patients are exposed to unnecessary risks of iatrogenic harms and healthcare systems are exposed to unnecessary expenditure resulting in significant opportunity costs.

Over the last 10 to 15 years, there has been increasing international policy and research attention on how to reduce the evidence-practice and policy gap. Across different healthcare systems, different terms describe these efforts including quality assurance, quality improvement, knowledge translation, knowledge utilisation, knowledge transfer and exchange, innovation diffusion, implementation research, research utilisation, evidence-informed policy, and evidence-informed health systems [4,5]. These different terms often cover related and overlapping constructs. Commenting on the terminology of quality assurance in 1982, Donabedian noted that ‘we have used these words in so many different ways that we no longer clearly understand each other when we say them’ [6]. Throughout this paper, we use the term ‘knowledge translation’ which has gained currency in Canada and globally over the last decade. There are two main types of translational research. T1 research refers to the translation of basic biomedical research into clinical science and knowledge, while T2 research refers to the translation of this new clinical science and knowledge into improved health [7]. In this paper, we refer to T2 research. We define knowledge translation as ‘ensuring that stakeholders are aware of and use research evidence to inform their health and healthcare decision-making.’ This definition recognizes that there are a wide range of stakeholders or target audiences for knowledge translation, including policy makers, professionals (practitioners), consumers (*i.e.*, patients, family members, and informal carers), researchers, and industry.

While knowledge translation is a relatively new term, the notion of moving research findings into practice is not new. It can be traced back to the investigations of French sociologist Gabriel Tarde at the beginning of the 20th century who attempted to explain why some innovations are adopted and spread throughout a society, while others are ignored [8]. The current conceptualization of knowledge translation evolved out of several diverse disciplinary perspectives, including knowledge utilisation, diffusion of innovations, technology transfer, evidence-based medicine, and quality improvement [9]. Interest in knowledge translation has increased dramatically in recent years due to recognition that traditional approaches to moving research into practice, which were predominantly based on education (*e.g.*, continuing professional development CPD), did not lead to optimal care. In this paper, based on a previously published monograph chapter [10], we summarise the current concepts and evidence to guide knowledge translation activities. We structure the article around five key questions identified by Lavis and colleagues [11]:

1. What should be transferred?

2. To whom should research knowledge be transferred?

3. By whom should research knowledge be transferred?

4. How should research knowledge be transferred?

5. With what effect should research knowledge be transferred?

## Discussion

### What should be transferred?

The increased focus on knowledge translation has frequently emphasised individual studies as the unit for knowledge translation. While this may be appropriate when the targets for knowledge translation are other researchers or research funders (who need to be aware of primary research results), we argue that this is inappropriate when the targets for knowledge translation are consumers, healthcare professionals, and/or policy makers. This is because individual studies rarely, by themselves, provide sufficient evidence for practice and policy changes. In fact, individual studies may be misleading due to bias in their conduct or random variations in their findings, although some exceptionally large randomised trials may be sufficiently persuasive by themselves to warrant practice or policy change, *e.g.*, the Antihypertensive and Lipid-Lowering Treatment to Prevent Heart Attack Trial (ALLHAT) [12] and the International Study of Infarct Survival 2 (ISIS-2) Trial [13].

Ioannidis and colleagues undertook a series of landmark studies of research exploring the evolution of evidence in healthcare (summarized in [14]). In both basic and clinical sciences, they observed the ‘Proteus phenomenon’—that the first published study on a scientific question may find the most extravagant effect size and that as further evidence is gathered, effect sizes tend to diminish [14]. They observed that thousands of observations were required before estimates of gene disease association became stable [15]. They also noted that the results of even highly cited clinical research studies published in major medical and specialty journals were likely to be contraindicated or found to be exaggerated with further accumulation of evidence [16]. As a result, Ioannidis and colleagues argued that replication and evidence synthesis is needed before knowledge translation [14].

We suggest that the results of individual studies need to be interpreted within the context of global evidence before deciding whether it is ready for knowledge translation. In other words, the basic unit of knowledge translation should be up-to-date systematic reviews or other syntheses of the global evidence. Greater emphasis on the results of systematic reviews would increase the ‘signal to noise’ of knowledge translation activities and may increase the likelihood of their success. Over the last twenty years, healthcare research funders and healthcare systems have made considerable investments in knowledge syntheses, especially those targeting the needs of healthcare practitioners and patients. Examples include the substantial number of publicly funded national guideline and health technology programs, The Cochrane Collaboration [17], and the US funded Evidence-based Practice Centers [18].

The question ‘What should be transferred?’ also challenges knowledge translators to identify the key messages for different target audiences and to fashion these in language and knowledge translation products that are easily assimilated by different audiences. Over the past decade, a variety of different products have been developed targeting different audiences (for example, decision aids for patients [19], clinical practice guidelines for healthcare professionals [20], and actionable messages [11] and policy briefs [21] for policy makers).

### To whom should research knowledge be transferred?

The relative importance of knowledge translation to different target audiences will vary by the type of research being translated. For example, primary target audiences for knowledge translation of the results of basic science include other researchers and industry; whereas primary target audiences for knowledge translation of the results of population health research include other researchers, administrators, and policy makers (See Table 1).

**Table 1:** Stakeholders for different types of research

**Potential stakeholder**	**Type of research**			
	**Basic**	**Clinical**	**Health Services**	**Population Health**
**Consumers**	**-**	**+++**	**+++**	**-**
**Professionals**	**-**	**+++**	**+++**	**-**
**Local Administrators**	**-**	**++**	**+++**	**+++**
**National Policy Makers**	**-**	**+++**	**+++**	**+++**
**Regulatory Bodies**	**+++**	**+++**	**+++**	**+++**
**Industry**	**+++**	**+++**	**++**	**+**
**Research Funder**	**+++**	**+++**	**+++**	**+++**
**Researchers**	**+++**	**+++**	**+++**	**+++**

Table Legend:

- Not Relevant.

+ Low Relevance to +++ High Relevance.

The relative importance of different target audiences will also vary by the results of the research [22]. For example, the primary target audiences for clinical research demonstrating lack of benefit or harms from a drug sufficient to warrant its withdrawal might be national policy makers (including regulatory bodies) and industry. Whereas, the primary target audiences for clinical research demonstrating benefits from a drug to suggest its widespread use might be patients, healthcare practitioners, local administrators as well as national policy makers, and industry (See Table 2).

**Table 2:** **Potential target audiences for clinical research about a drug** (adapted from Mowatt *et al.*, 1998 [22])

	**Stop use**	**Stop use, promote research**	**Promote use for limited indications**	**Promote use for new indication**	**Promote widespread use**
**Consumers**	S	S	P	S	P
**Healthcare Professionals**	S	S	P	P	P
**Administrators**	S	S	P	P	P
**National Policy Makers**	P	P	P	P	P
**Regulatory Body**	P	P	P	P	P
**Industry**	P	P	P	P	P
**Research Funders**		P			
**Researchers**		P			

Table Legend:

P = Primary Target Audience.

S = Secondary Target Audience.

### By whom should research knowledge be transferred?

The messenger in knowledge translation efforts may be an individual (*e.g.*, healthcare practitioner, researcher, or consumer), group, organization, or even healthcare system. The most appropriate messenger will vary according to the target audience and research knowledge being transferred. Shonkoff suggests that in determining ‘who’ should be the messenger credibility is important [23]. Research supports this view; Hayward and colleagues found that an authoritative endorsement by a respected physician organization or physician colleague influenced physicians’ use of clinical practice guidelines in practice [24]. With public policy makers, Lavis and colleagues suggest that the most credible messengers might include organizations of government officials [11].

Building credibility and acting as a messenger for the transfer of research knowledge is a time-consuming and skill-intensive process, making it impossible to use a ‘one size fits all’ approach to deciding ‘by whom should research knowledge be transferred’ [11]. Researchers typically carry the responsibility for conducting knowledge translation. They should, however, only be the messenger when they have credibility with the target audience, possess the skills and experience needed to transfer the research knowledge at hand, and have time and resources to do so. A more appropriate approach to effective and sustainable knowledge translation may be the development of research knowledge infrastructures by healthcare systems that address the needs of their various stakeholders (*e.g.*, consumers, practitioners, managers, and policy makers). Ellen and colleagues define research knowledge infrastructure as any instrument (*i.e.*, programs, tools, devices) implemented in a healthcare system in order to facilitate access, dissemination, exchange, and/or use of evidence [25]. Components of research knowledge infrastructures are classified into two broad categories: technological and organizational. Technological components include electronic databases and search engines. Organizational components include documentation specialists, data analysts, knowledge brokers (*i.e.*, individuals who manage the collaboration between an organization, external information, and knowledge producers and users), and training programs (to assist with activities such as searching for information, quality appraisal, adaption and use of the research findings) [25,26].

In Canada, some knowledge translation researchers have invested significant time and financial resources into building technological (online databases with built-in search engines) resources that can be used by healthcare systems as part of a research knowledge infrastructure. *Rx for Change* is an online database that houses syntheses of the global evidence from systematic reviews: on the effectiveness of interventions for improving prescribing by healthcare professionals and medicines use by consumers; of professional interventions that impact the delivery of care; and of organizational, financial, and regulatory interventions that influence professional behaviour. The methods used to populate the database parallel systematic-review methodology. *Rx for Change* is publicly accessible and contains research information relevant to healthcare professionals, consumers, policy makers, and researchers [27].

*Health Systems Evidence* is also an online database, but primarily targets policy makers and senior managers (and other individuals responsible for assisting or making public policy decisions). Common criticisms of systematic reviews by policy makers include the absence of relevant reviews, and difficulty accessing and understanding reviews. *Health Systems Evidence* addresses these criticisms in order to facilitate the use of systematic reviews in health systems and policy decision making. *Health Systems Evidence* is a repository of syntheses of research evidence about governance, financial, and delivery arrangements within health systems, and about implementation strategies that can support change in health systems. The database contains policy briefs, overviews of systematic reviews, systematic reviews, and soon will contain a range of other types of documents needed in the policymaking process, such as economic evaluations.

Both databases (*Rx for Change* and *Health Systems Evidence*) provide improved access to research information for consumers, practitioners, and/or policy makers. However, this access is necessary but not sufficient to ensure knowledge translation. Effective and sustainable knowledge translation also requires organizational knowledge infrastructure components. Ellen and colleagues developed a research knowledge infrastructure framework that identified potential organizational components that a healthcare system could have in its research knowledge infrastructure. This framework is based on an environmental scan and scoping review of existing literature. The broad organizational domains included in the framework are: climate for research use, research production, activities used to link research to action including push efforts (*i.e.*, efforts undertaken by researchers to disseminate research evidence to knowledge users), pull efforts (*i.e.*, efforts by knowledge users to access and use research evidence), and exchange efforts (*i.e.*, efforts focused on building and maintaining relationships between researchers and knowledge users), and evaluation of efforts [25]. This framework is currently being evaluated in a study examining knowledge-translation platforms in 41 countries [25,28].

### How should research knowledge be transferred?

#### Planning for knowledge translation

There are a large number of planned knowledge translation models, derived from different disciplinary and contextual viewpoints [29,30]. Most of these suggest that planned knowledge translation is more likely to be successful if an assessment of the likely barriers and facilitators informs the choice of knowledge translation strategy. In this section, we briefly discuss types of barriers, potential approaches for identifying barriers, and factors influencing the choice of knowledge translation intervention.

#### Identifying barriers to knowledge translation

Common barriers across target groups include issues relating to knowledge management, such as the sheer volume of research evidence currently produced, access to research evidence sources, time to read evidence sources and skills to appraise and understand research evidence. Over the past twenty years, there has been substantial investment by many healthcare systems to address these knowledge management barriers. For example, the conduct of systematic reviews and development of clinical practice guidelines to reduce the volume of research evidence and the time needed to read evidence sources; investment in electronic libraries of health and public access evidence sources to improve access to research evidence; and the development of critical appraisal skills tools and training to improve research literacy skills.

However while better knowledge management is necessary, it is unlikely by itself to be sufficient to ensure knowledge translation because of barriers working at different levels of healthcare systems, many of which operate at levels beyond the control of an individual practitioner. For example, barriers may operate at other levels of a healthcare system including: structural barriers (*e.g.* financial disincentives), organizational barriers (*e.g.* inappropriate skill mix, lack of facilities or equipment), peer group barriers (*e.g.* local standards of care not in line with desired practice), professional (*e.g.* knowledge, attitudes and skills) and professional-patient interaction barriers (*e.g.* communication and information processing issues). Evidence in support of this can be found in a structured review of healthcare professionals’ views on engagement in quality improvement activities [31]. In this review, Davies and colleagues concluded that many of the barriers to participating in quality improvement activities identified by professionals arise from problems related to working effectively between and across health professions. This means that although knowledge management resources (*e.g.*, more time and more resources) may be necessary and even helpful, they are unlikely to be sufficient to overcome the other ‘organizational’ barriers professionals face to engage in quality improvement (and knowledge translation) activities [31].

There are diverse methods for identifying potential barriers including qualitative approaches (individual interviews, focus groups), surveys and direct observation [32]. However, there are no standard approaches available yet. As a result, those involved with knowledge translation activities need to use their judgement about how best to elicit barriers given their understanding of the context and potential barriers and resources available to them.

#### Choosing interventions

Unfortunately, our evidence on the likely effectiveness of different strategies to overcome specific barriers to knowledge translation remains incomplete. Individuals involved in knowledge translation need to: identify modifiable and non-modifiable barriers relating to behavior; identify potential adopters and practice environments; and prioritise which barriers to target based upon consideration of ‘mission critical’ barriers. Furthermore, the potential for addressing these barriers through knowledge translation activities (based upon consideration of the likely mechanisms of action of interventions) and the resources available for knowledge translation activities also needs to be addressed.

#### Effectiveness of professional behaviour change strategies

The Cochrane Effective Practice and Organisation of Care (EPOC) group supports reviews of interventions to improve healthcare systems and healthcare delivery [33]. It has identified over 7,000 randomised and quasi-experimental studies and conducted 80 systematic reviews of professional, organisational, financial, and regulatory interventions within its scope by August 2011.

EPOC has prepared two overviews of systematic reviews and is currently updating these [34,35]. It has identified over 300 systematic reviews of professional behaviour change strategies. In this section, we summarise the results of key Cochrane EPOC reviews, selected because they are in general of higher quality and more up-to-date than non-Cochrane systematic reviews of similar focus [36]. We provide a definition of each intervention, the likely mechanism of action of the intervention, and any comments relating to the practical delivery of the intervention (including the resources required). The details and findings of the reviews of the interventions, including the median and range of effect sizes observed, are presented in Table 3.

**Table 3:** Effectiveness of professional behaviour change strategies from selected EPOC systematic reviews

**Intervention**	**Number of studies/individuals**	**Effect sizes**
**Review**		
**Printed Educational Materials**	12 randomised trials	Median absolute improvement of care on categorical process outcomes (*e.g.*, x-ray requests, prescribing and smoking cessation activities) of 4.3% (range −8.0% to +9.6%) across studies.
	11 nonrandomised studies	
Farmer *et al.*[37]		
**Educational Meetings**	81 randomised trials (involving more than 11,000 health professionals)	Median absolute improvement in care of 6.0% (interquartile range +1.8% to 15.3%).
Forsetlund *et al.*[38]		
		Larger effects were associated with higher attendance rates, mixed interactive and didactic meetings and interactive meetings.
		Smaller effects were observed for complex behaviours and for less serious outcomes.
**Educational Outreach**	69 randomised trials (involving more than 15,000 health professionals)	Median absolute improvements in:
		·prescribing behaviours (17 comparisons) of 4.8% (interquartile range +3.0% to + 6.5%);
O’Brien *et al.*[39]		
		·other behaviours (17 comparisons) of 6.0% (interquartile range +3.6% to +16.0%).
		The effects of educational outreach for changing more complex behaviours are less certain.
**Local Opinion Leaders**	18 randomised trials (involving more than 296 hospitals and 318 primary care physicians)	Median absolute improvement of care of 12.0% across studies (interquartile range +6.0% to +14.5%).
Flodgren *et al.*[40]		
**Audit and Feedback**	118 randomised trials	Median absolute improvement of care of 5.0% (interquartile range +3% to +11%).
Jamtvedt *et al.*[41]		
		In general, larger effects were seen if baseline compliance was low.
**Computerized Reminders**	28 randomised trials	Median absolute improvement of care of 4.2% (interquartile range +0.8% to +18.8%).
Shojania *et al.*[42]		
		Comment: Most studies have examined the effects of relatively simple reminders; the results of more complex decision support systems, especially for chronic disease management, have been less successful.
**Tailored Interventions**	26 randomised trials	Meta-regression using 12 randomised trials. Pooled odds ratio of 1.52 (95% CI, 1.27 to 1.82, p < .001)
Baker *et al.*[43]		

Generally, similar median absolute effect sizes are reported across the interventions. While one interpretation might be that the choice of intervention is less important than doing something/anything (*i.e.*, that the observed effects are largely non specific (Hawthorne-like) effects), we do not believe this to be the case. The interquartile range of absolute effect sizes covers almost 30 percentage points and varies by intervention (see Table 3). Furthermore, the variation in observed effects within intervention category (for example the interquartile range of observed effects in trials of audit and feedback was +3% to +11% absolute improvements in performance) suggest that the effects of interventions vary presumably related to the degree to which the mechanism of action of the intervention addresses the underlying barriers in a study. The interventions also have very different mechanisms of action, and there is likely to be confounding within and across reviews. In other words, researchers are likely to have tested interventions that they believed likely effective given the particular behaviours and likely barriers within the context of their study. Finally, because we are reporting absolute effects some broad commonality of effect sizes is to be expected. In general, interventions are not tested in the expectation of producing large absolute effect sizes. Most cluster trials are powered to detect effects in the range of 10 to 20 percent absolute improvement. Under these circumstances similarity of observed effects is not surprising.

#### Printed educational materials

EPOC defines printed educational materials as the ‘distribution of published or printed recommendations for clinical care, including clinical practice guidelines, audio-visual materials and electronic publications. The materials may have been delivered personally or through mass mailings’ [37]. In general, printed educational materials target knowledge and potential skill gaps of individual healthcare professionals. While they could also be used to target motivation when written as a ‘persuasive communication’ there is little evidence of them being used in this way. Printed educational materials are commonly used, have a relatively low cost and are generally feasible in most settings.

#### Educational meetings

EPOC defines educational meetings as the ‘participation of healthcare providers in conferences, lectures, workshops or traineeships’ [38]. An important distinction is between didactic meetings (that largely target knowledge barriers at the individual healthcare professional/peer group level) and interactive workshops (that can target knowledge, attitudes, and skills at the individual healthcare professional/peer group level). Educational meetings are commonly used, with the main cost related to the release time for healthcare professionals, and are generally feasible in most settings.

#### Educational outreach

EPOC defines educational outreach or academic detailing as ‘use of a trained person who meets with providers in their practice settings to give information with the intent of changing the providers’ practice. The information given may have included feedback on the performance of the provider(s)’ [39]. Soumerai and Avorn suggest that educational outreach derives from social marketing approaches that target an individual’s knowledge and attitudes [44]. Typically, the detailer aims to get a maximum of three messages across during a 10 to 15 minute meeting with a healthcare provider. The detailer will tailor their approach to the characteristics of the individual healthcare provider, and typically use additional provider behaviour change strategies to reinforce their message. Most studies of educational outreach have focused on changing relatively simple behaviours in the control of individual physician behaviors such as the choice of drugs to prescribe.

Educational outreach programs have been used across a wide range of healthcare settings especially to target prescribing behaviours. They require considerable resources including the costs of detailers and preparation of materials. Nevertheless, Mason and colleagues observed that educational outreach may still be efficient to change prescribing patterns [45].

#### Local opinion leaders

EPOC defines local opinion leaders as ‘use of providers nominated by their colleagues as ‘educationally influential’ [40]. The investigators must have explicitly stated that their colleagues identified the opinion leaders.’ Opinion leadership is the degree to which an individual is able to influence other individuals’ attitudes or overt behaviour informally in a desired way with relative frequency. This informal leadership is not a function of the individual’s formal position or status in the system; it is earned and maintained by the individual’s technical competence, social accessibility, and conformity to the systems norms. When compared to their peers, opinion leaders have greater exposure to all forms of external communication, have somewhat higher social status and are more innovative. However, the most striking feature of opinion leaders is their unique and influential position in their system’s communication structure; they are at the centre of interpersonal communication networks (interconnected individuals who are linked by patterned flows of information). Opinion leaders target the knowledge, attitudes, and social norms of their peer group. The potential success of opinion leaders is dependent upon the existence of intact social networks within professional communities. Grimshaw and colleagues observed that the existence of such networks varied across communities and settings within the UK [46]. They also observed that opinion leaders were condition-specific; in other words, colleagues identified different opinion leaders for different clinical problems. Doumit also observed that opinion leaders where not stable over time [47]. The resources required for opinion leaders include costs of the identification method, training of opinion leaders and additional service costs.

#### Audit and feedback

EPOC defines audit and feedback as ‘any summary of clinical performance of healthcare over a specified period of time’ to change health professional behaviour, as indexed by ‘objectively measured professional practice in a healthcare setting or healthcare outcomes.’ The summary may also have included recommendations for clinical action. The information may have been obtained from medical records, computerised databases, or observations from patients. The subsequent feedback of and resulting action planning based on the audit summary are also important elements of an audit and feedback intervention [41,48]. Adams and colleagues observed that healthcare professionals often over estimated their performance by around 20% to 30% [49]. Audit and feedback target healthcare provider/peer groups’ perceptions of current performance levels and is useful to create cognitive dissonance within healthcare professionals as a stimulus for behaviour change. The resources required to deliver audit and feedback include data abstraction and analysis costs and dissemination costs. The feasibility of audit and feedback may depend on the availability of meaningful routine administrative data for feedback.

#### Reminders

EPOC defines reminders as ‘patient or encounter specific information, provided verbally, on paper or on a computer screen, which is designed or intended to prompt a health professional to recall information [42]. This would usually be encountered through their general education, in the medical records or through interactions with peers, and so remind them to perform or avoid some action to aid individual patient care. Computer aided decision support and drugs dosage are included.’ Reminders prompt healthcare professionals to remember to do important items during professional-patient interactions [50]. The majority of early studies on computerized reminders were undertaken in highly computerized US academic health science centres, and their generalisability to other settings is less certain [51]. The resources required vary across the delivery mechanism. Additionally, there is insufficient knowledge at present about how to prioritise and optimize reminders.

#### Tailored interventions

Tailored interventions are ‘strategies to improve professional practice that are planned taking account of prospectively identified barriers to change’ [43]. Barriers to change refer to factors that have the potential to impair the effectiveness of interventions designed to improve professional behaviour/practice. EPOC classifies barriers to change into nine categories (information management, clinical uncertainty, sense of competence, perceptions of liability, patient expectations, standards of practice, financial disincentives, administrative constraints, and other) [52]. In a recent review, Baker and colleagues assessed the effectiveness of interventions tailored to address identified barriers to change on professional practice or patient outcomes and found that tailored interventions are more likely to improve professional practice (*e.g.*, prescribing and adherence to guideline recommendations) than is no intervention or the dissemination of guidelines or educational materials. Further research is needed to determine the effectiveness of tailored interventions in comparison with other interventions [43].

#### Multifaceted interventions

**Figure 1:**
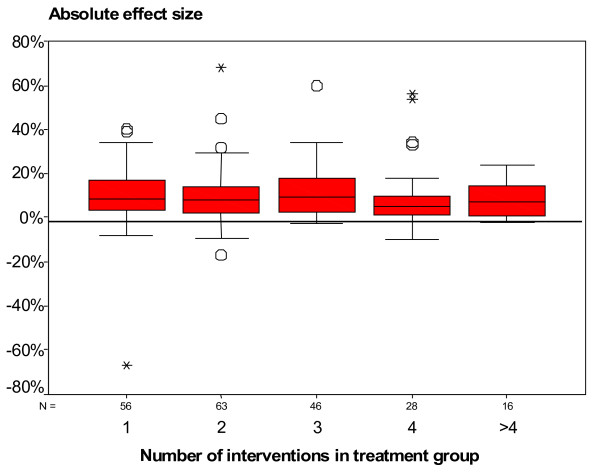
Effect sizes of multifaceted interventions by number of interventions.

EPOC defines multifaceted interventions as ‘any intervention including two or more components.’ Multifaceted interventions potentially target different barriers in the system. Grimshaw and colleagues explored whether there was a dose response curve for multifaceted interventions and observed that effect sizes did not necessarily increase with increasing number of components (Figure 1) [20]. They also observed that few studies provided any explicit rationale or theoretical base for the choice of intervention. As a result, it was unclear whether researchers had an *a priori* rationale for the choice of components in multifaceted interventions based upon possible causal mechanisms or whether a ‘kitchen sink’ approach formed the basis for the choice. It is plausible that multifaceted interventions built upon a careful assessment of barriers and coherent theoretical base may be more effective than single interventions. Multifaceted interventions are likely to be more costly than single interventions. When planning multifaceted interventions, it is important to carefully consider how components are likely to interact to maximise benefits.

#### Effectiveness of knowledge translation strategies focusing on consumers

The Cochrane Consumers and Communication Review Group supports systematic reviews of the effects of interventions (particularly those which focus on information and communication) which affect consumers’ interactions with healthcare professionals, healthcare services and healthcare researchers [53]. Outcomes of interest include effects on people’s knowledge and decision-making, healthcare use, experience of healthcare, and health and wellbeing. They have identified over 7,000 randomised studies and conducted 35 systematic reviews of interventions and one overview of systematic reviews [54] within their scope to August 2011.

The Cochrane Consumers and Communication Review Group have developed a taxonomy for organising interventions. Categories relevant to knowledge translation include interventions: to facilitate communication and/or decision making; to support behaviour change; and to inform and educate. In this section, we summarize the range of intervention types relevant to knowledge translation by consumers. Drawing from the Cochrane reviews, we present the authors’ definition of each intervention; the details and findings of the reviews are presented in Table 4.

**Table 4:** Effectiveness of knowledge translation strategies focusing on consumers from selected systematic reviews

**Intervention**	**Number of studies**	**Findings (Effectiveness)**
**Review**		
**Decision Aids**	86 randomised trials (involving more than 20,209 participants)	Compared with usual care, decision aids:
Stacey *et al.*[55]		·improved knowledge and accuracy of risk perceptions;
		·reduced the proportion of people who were passive in decision-making;
		·resulted in a higher proportion of patients achieving decisions informed and consistent with their values (when decision aids included an explicit values clarification component);
		·reduced the number of people remaining undecided;
		·reduced decisional conflict;
		·decreased the choice of major elective surgery in favour of conservative options.
		Decision aids have no adverse effects on satisfaction but further research is needed to clarify their effect on adherence to chosen option, patient-practitioner communication, cost-effectiveness and use with developing or lower literacy populations.
**Personalised Risk Communication**	22 randomised trials	There was weak evidence, consistent with a small effect, that personalised risk communication (whether written, spoken or visually presented) increases uptake of screening tests.
Edwards *et al.*[56]		
**Communication before Consultations**	33 randomised trials (involving 8244 participants)	Compared with a control, communication before consultations increased question asking during consultations. They may also increase patient participation in consultation and improve patient satisfaction.
Kinnersley *et al.*[57]		
		Both coaching and written material interventions produced similar effects on question asking, but coaching produced a larger increase in patient satisfaction.
		Overall the benefits of ‘communication before consultations’ interventions were minor.
**Interactive Health Communication Applications (IHCAs)** (2 reviews)		
Murray *et al.*[58]	24 randomised trials (involving 3739 participants)	IHCAs had a significant positive effect on knowledge, social support and clinical outcomes.
Bailey *et al.*[59]	15 randomised trials (involving 3917 participants)	Positive effects of IHCAs on knowledge, safer sex self-efficacy and intentions and sexual health behavior were found.
		Comment: Data were insufficient for meta-analysis of biological outcomes or analysis of cost- effectiveness and thus, the effects on these outcome categories remain unknown.
**Interventions to Enhance Medication Adherence**	78 randomised trials	Mixed effects were observed for short term and long- term medication adherence.
Haynes *et al.*[60]		Some, but not all, of the simple interventions, such as counselling, written information and personal phone calls, were effective with people on short-term medication treatments.
		The picture for the effectiveness of interventions for longer-term treatments was mixed; few interventions showed promise and those that were effective were complex and multifaceted in nature.
**Contracts**	30 randomised trials (involving 4691 participants)	Contracts were shown to ‘potentially’ improve patient adherence (as applied to diagnostic procedures, therapeutic regimens, and/or a health promotion or illness prevention initiative).
Bosch-Capblanch *et al.*[61]		
		Comment: The result above is based on only half of the included studies; the effects were not detected over longer periods.
**New Methods of Communication**		
(2 reviews)		
Marteau *et al.*[62]	13 randomised trials	Little or no effect was shown with respect to smoking cessation or increasing physical activity. A small effect was shown for changing diet.
	(on communicating DNA-based disease risk estimates)	
		The intervention showed potential for altering intentions to change behaviour (in six non-clinical analogue studies).
		Comment: The authors concluded that given the small number of trials in this area, more research involving ‘better-quality RCTs’ is needed before recommending application in practice.
Hollands *et al.*[63]	9 randomised trials (involving 1371 participants)	Overall, results were mixed:
		·a positive effect was found for smoking cessation (three trials);
		·a positive effect was found for skin examination behaviour (one trial);
	(providing visual feedback on medical imaging results)	·no effect was found for change in physical activity (one trial).
		Comment: The authors concluded that due to the small number of trials and the mixed results found, the effectiveness of communicating medical imaging results to change health behaviour is largely unknown and thus, its application in practice is not yet recommended.
**Written Information** Nicolson *et al.*[64]	25 randomised trials (involving 4788 participants)	Written material significantly improved knowledge of medicines in six of twelve trials. In three of these six trials recall of side effects also improved, but medicines recall significantly improved in only a minority of trials (one of four).
		The results for attitudinal and behavioural outcomes were mixed.
		Comment: Overall, the authors concluded the combined evidence from this review is not sufficient to say whether written medicines information is effective in changing behaviours related to medicine taking.
**Self Management Programmes** Foster *et al.*[65]	17 randomised trials (involving 7442 participants)	Small (clinically insignificant) short-term improvements in pain, disability, fatigue and depression were found.
	(Self management programmes run by lay people)	Positive effects on confidence to manage and self- rated health were also found.
		There was no effect on quality of life or use of health services.

#### Interventions to facilitate communication and/or decision-making

Three interventions to facilitate communication and/or decision making that have been the focus of Cochrane systematic reviews are decision aids, personalised risk communication, and communication before consultations. Decision aids are a type of decision support intervention designed to help people make choices about health treatment options. Stacey (following O’Connor, who prepared the first Cochrane review), defines them as interventions containing ‘detailed, specific, and personalized information to help people focus on options and outcomes for the purpose of decision making’ [55]. They are important for decisions where there is uncertainty about a specific course of action. Personalised risk communication refers to the provision of information to consumers that is personally relevant to them. It is sometimes used to present and discuss the risks and benefits of healthcare in general, and of screening in particular, to consumers. As Edwards and colleagues outline, it can be based on a consumer’s own risk factors for a condition (*e.g.*, their age) or calculated from their risk factors using epidemiological formulas. In the latter, the information is often presented as an absolute risk or as a risk score, or categorised into, for example, high-, medium-, or low-risk groups. Personalised risk communication may also be less detailed, for example, a listing of a consumer’s risk factors to guide discussion and intervention [56]. In their Cochrane review, Kinnersley and colleagues operationalise communication before consultations to include any intervention delivered before consultations, and which has been designed to help consumers (and/or their representatives) address their information needs within consultations [57].

#### Interventions to support behaviour change

One area that continues to challenge the Cochrane Consumers and Communication Review Group is the identification of effective interventions that support behaviour change. Four interventions which have been the focus of Cochrane reviews in this area are: interactive health communication applications; interventions to enhance medication adherence; contracts; and new methods of communication. Interactive health communication applications, defined by Murray and colleagues, are computer-based (usually web-based) information packages for patients that combine health information with at least one of: social support, decision support, or behaviour change support [58]. Interventions to enhance medication adherence include a wide range of single and multifaceted interventions; Haynes and colleagues identified: instruction, counseling, automated telephone monitoring and counseling, manual telephone follow-up, family intervention, increasing the convenience of care, simplified dosing, self-monitoring, reminders, special ‘reminder’ pill packaging, dose-dispensing units and medication charts, appointment and prescription refill reminders, reinforcement/rewards, medication formulations, crisis intervention, direct observation of treatments, lay health mentoring, comprehensive pharmaceutical care services, and psychological therapy in their Cochrane review [60]. Contracts refer to formalised (written or verbal) mutual agreements between two or more parties [61]. New methods of communication to date have included communicating DNA-based disease risk estimates to change health behaviours on lifestyle (*e.g.*, smoking, physical activity, diet) [62] and providing consumers with a visual presentation (*i.e.*, the source images) of their medical imaging (*i.e.*, of magnetic resonance imaging, tomography, radiography, and/or ultrasonography) results to increase consumers’ engagement in health-related behaviours [63].

#### Interventions to inform and educate

Two interventions which have been the focus of Cochrane reviews to ‘inform and educate’ consumers are written information and self-management programmes. Written information is one of the most ubiquitous interventions targeting consumers [64].

Self management programmes have become a major initiative of government and community organizations in the area of chronic illness [65]. They promote various strategies for people to take an active approach to managing their health.

#### Effectiveness of knowledge translation strategies focusing on policy makers and senior health service managers

In contrast to the substantial evidence base on the effectiveness of knowledge translation strategies targeting healthcare professionals and consumers, few systematic reviews exist of interventions evaluating the effects of knowledge translation strategies for policy makers or senior health service managers. One review, conducted by Perrier and colleagues, evaluated interventions to increase the use of systematic reviews by health policy makers and managers [66]. Two studies were included in the review. The first study utilized a non-experimental design to report an intervention where public health policy makers were offered the opportunity to receive five relevant reviews. At three months and two years, respectively, 23% and 63% of respondents reported using at least one of the systematic reviews to make a policy decision. The second study was a randomised trial where health departments received one of three interventions: access to an online registry of systematic reviews, tailored messages plus access to the online registry of systematic reviews, or tailored messages plus access to the registry along with a knowledge broker who worked one-on-one with decision makers over a period of one year. While none of the interventions showed a significant effect on global evidence-informed decision making, tailored messages plus access to the online registry of systematic reviews showed a positive significant effect on public health policies and programs [66].

Lavis and colleagues conducted a systematic review of factors that influence the use of research evidence in public policy making [67]. Five criteria were used to assess validity of the included studies: the use of two or more data collection methods; a random or purposive sampling strategy; response rate >60%; two or more types of research use are examined; and two or more competing variables are examined.

A total of 16 studies met the criteria of using two or more data collection methods. These studies were conducted across a variety of jurisdictions, policy domains, content areas, and time periods. There was relatively little consistency in findings. However, two factors emerged with some frequency as being important to policy makers’ use of research evidence: interactions between researchers and policy makers in the context of policy networks such as formal advisory committees and in the context of informal relationships; and research that matched the beliefs, values, interests, or political goals and strategies of elected officials, social interest groups, and others. Both factors increased the prospects for research use by policy makers [67].

The findings from these reviews and other findings have led to the development of a number of knowledge translation approaches targeting policy makers and senior health services managers [28,68,69]. For example, a series of tools called *SUPPORT Tools for evidence-informed health policy making (STP)* were developed to assist policy makers in using research evidence. These tools were developed by members of *the SUPporting POlicy relevant Reviews and Trials (SUPPORT) project,* an international collaboration funded by the European Commission’s 6th Framework [70] (http://www.supportcollaboration.org). The SUPPORT tools describe a series of processes to help ensure that relevant research is identified, appraised and used appropriately by policy makers. The tools address four broad areas of interest related to policymaking: supporting evidence-informed policymaking [71-73]; identifying needs for research evidence in relation to clarifying problems, framing options, and planning implementation [74-76]; finding and assessing evidence from systematic reviews [77-79] and other kinds of evidence [80,81]; and moving from research evidence to decisions. The focus in this final area is on engaging stakeholders in evidence-informed policymaking [21,82,83] and on addressing how to use research evidence in decisions [84-86]. By focusing on how to ‘support’ the use of research evidence in health policymaking, the SUPPORT tools should increase the use of research evidence by policy makers [87]*.*

The SUPPORT tools describe a variety of packaging and push, facilitating pull, and exchange activities. Packaging and push activities focus on the activities of researchers to disseminate their research findings to a broad audience above and beyond traditional routes of dissemination such as publication in peer reviewed journals [11]. Examples of packaging and push activities include: increased emphasis on knowledge syntheses as the unit for knowledge translation; actionable messages; graded entry formats to allow the research user to access the level of detail that he or she requires (for example, the Canadian Health Services Research Foundation requires research reports to have one page of main messages, a three-page executive summary, and then no more than 25 pages for the complete project); using multiple communication channels tailored to the target audience; targeted electronic push of information relevant to the specific needs of research users—examples include the Contacts, Help, Advice and Information Network (C.H.A.I.N.) ([88], http://chain.ulcc.ac.uk/chain/ accessed 5 July 2011) and E-watch bulletin on Innovation in Health Services (http://www.ohpe.ca/node/2740 accessed 5 July 2011); workshops and seminars with target audiences; and development of tools to help research users apply research findings in their own settings.

Facilitating pull activities focus on the needs of users, and creating an appetite for research results [11]. Pull activities include various training activities to improve policy makers’ and senior managers’ research literacy and interest. For example, the Canadian Health Services Research Foundation provides the EXTRA program to train senior healthcare executives in research application (http://www.chsrf.ca/Programs/EXTRA.aspx accessed 5 July 2011). ‘One stop’ initiatives such as Health Systems Evidence also facilitate pull.

Exchange activities focus on building and maintaining new relationships between researchers and policy makers and senior managers to exchange knowledge and ideas [69,89]. For example, several research-funding programs require active participation of decision makers (sometimes including co-funding by healthcare organisations) in research teams. The rationale is that decision makers are more likely to consider research findings if they are actively involved in the research conducted in their settings to answer specific contextualized questions. These approaches legitimately focus on local knowledge translation of individual studies. However, the results of these studies should still be incorporated into systematic reviews to judge whether additional knowledge translation activities should be undertaken outside the context and relationships of the original study. Other exchange approaches include deliberative dialogues and the use of knowledge brokers to act as ‘human intermediaries’ between the world of research and action [69,82,90].

This profusion of approaches to improving knowledge translation to policy makers and senior healthcare managers highlights the increased recognition of the failure of traditional diffusion approaches to knowledge translation for this target group (*e.g.*, [90]). Most of these approaches have a strong theoretical basis and face validity. However, it will be important to evaluate their benefits, harms and costs fully.

#### With what effect should research knowledge be transferred?

Appropriate endpoints of knowledge translation may vary across different stakeholder groups. For example, knowledge translation targeting policy makers and consumers should ensure that consideration of research evidence is a key component of their decision making, but recognize that there are other legitimate factors (for example, the policy context for policy makers, values and preferences of individual patients) that need to considered [91-93]. Thus, the resulting decision is likely to be evidence-informed but may not be particularly evidence-based. However, knowledge translation targeting professionals should result in practice that is more evidence-based and is likely to be observable as reflected in changes in professional behaviours and quality indicators.

## Summary

In this paper, we have attempted to briefly summarise some of the key concepts and evidence about the effectiveness of knowledge translation activities targeting different stakeholder groups. We particularly recommend the five key questions developed by Lavis and colleagues as an aide for researchers and others involved in knowledge translation when developing knowledge translation activities [11]. There is a substantial (if incomplete) evidence base to guide choice of knowledge translation activities targeting healthcare professionals and patients. The evidence base on the effects of different knowledge translation approaches targeting healthcare policy makers and senior managers is much weaker but there are a profusion of innovative approaches that warrant further evaluation.

Grol observed that many current knowledge translation activities are based on participants’ beliefs, rather than evidence about the likely effectiveness of different approaches [94]. Grol challenged healthcare systems to develop and use a robust evidence base to support the choice of knowledge translation strategies, arguing, ‘evidence-based medicine should be complemented by evidence-based implementation.’ While we are some way from achieving this goal, there are grounds for optimism. Over the past twenty-five years, healthcare systems have invested heavily in knowledge synthesis activities that facilitate timely access of evidence. Further, it is possible to achieve clinically important practice changes by healthcare professionals and improved patient decision making with current knowledge translation activities.

## Competing interests

MPE is Co-Editor in Chief and JMG and JNL are Editorial Board members of Implementation Science. All editorial decisions on this manuscript were made by another editor.

## Authors’ contributions

JMG conceived of the idea of the paper. All authors contributed to writing the manuscript and approved the final version.
